# Pharmacokinetics of afatinib in subjects with mild or moderate hepatic impairment

**DOI:** 10.1007/s00280-014-2484-y

**Published:** 2014-06-07

**Authors:** David Schnell, Susanne Buschke, Holger Fuchs, Dietmar Gansser, Rainer-Georg Goeldner, Martina Uttenreuther-Fischer, Peter Stopfer, Sven Wind, Marc Petersen-Sylla, Atef Halabi, Rüdiger Koenen

**Affiliations:** 1 Translational Medicine and Clinical Pharmacology, Boehringer Ingelheim Pharma GmbH & Co KG, Biberach, Germany; 2 Drug Metabolism and Pharmacokinetics, Boehringer Ingelheim Pharma GmbH & Co KG, Biberach, Germany; 3 Global Biometrics and Clinical Applications, Boehringer Ingelheim Pharma GmbH & Co KG, Biberach, Germany; 4 Clinical Development and Medical Affairs, Boehringer Ingelheim Pharma GmbH & Co KG, Biberach, Germany; 5 Clinical Research Services (CRS), Kiel GmbH, Kiel, Germany

**Keywords:** Afatinib, Hepatic impairment, Pharmacokinetics, Human, Epidermal growth factor receptor (EGFR), Tyrosine kinase inhibitor

## Abstract

**Purpose:**

Afatinib, an oral irreversible ErbB family blocker, undergoes minimal metabolism by non-enzyme-catalysed adduct formation with proteins or nucleophilic small molecules and is predominantly non-renally excreted via the entero-hepatic system. This trial assessed whether mild or moderate hepatic impairment influences the pharmacokinetics of afatinib.

**Methods:**

This was an open-label single-dose study. Pharmacokinetic parameters after afatinib 50 mg were investigated in subjects with mild (*n* = 8) or moderate (*n* = 8) hepatic impairment (Child-Pugh A and B) and healthy controls (*n* = 16) matched for age, weight and gender. Plasma and urine samples for pharmacokinetic assessment were collected before and up to 10 days after dosing. Additional blood samples were drawn to determine ex vivo plasma protein binding of afatinib. Primary endpoints were comparisons of afatinib *C*_max_ and AUC_0**–**∞_ between subjects with hepatic impairment and healthy matched controls. Study progression was based on drug-related toxicity (CTCAE v. 3.0) and *C*_max_ of afatinib.

**Results:**

Afatinib pharmacokinetic profiles and plasma protein binding were similar in subjects with impaired liver function and healthy controls. Compared with matched controls, the afatinib-adjusted geometric mean ratio for AUC_0**–**∞_ was 92.6 % (90 % CI 68.0–126.3 %) and *C*_max_ was 109.5 % (90 % CI 82.7–144.9 %) for subjects with mild hepatic impairment, and 94.9 % (90 % CI 72.3–124.5 %) and 126.9 % (90 % CI 86.0–187.2 %), respectively, for subjects with moderate hepatic impairment. For all parameters, the 90 % CI included 100 %. Afatinib was generally well tolerated with no serious adverse events reported.

**Conclusion:**

Mild to moderate hepatic impairment had no clinically relevant effect on the pharmacokinetics of a single 50 mg dose of afatinib, implying that adjustments to the starting dose of afatinib are not considered necessary in this patient population.

## Introduction

Afatinib is an oral irreversible ErbB family blocker being investigated as a potential treatment for a variety of solid tumours [1], including epidermal growth factor receptor (EGFR)-mutation-positive non-small cell lung cancer (NSCLC), and metastatic head and neck cancer [2–6]. It was recently approved in various countries for the treatment of locally advanced or metastatic NSCLC with activating EGFR mutation(s). Afatinib selectively and potently blocks signalling from all relevant ErbB family dimers: EGFR (ErbB1), human EGFR-2 (HER2; ErbB2) and ErbB4 [1, 7]. Afatinib also blocks transphosphorylation of ErbB3 [7].

In patients with various solid tumours, peak plasma concentrations (*C*_max_) of afatinib are achieved 2–5 h after dosing [8]. Afatinib exhibits at least bi-exponential disposition with steady state reached within 8 days. Afatinib displays a high apparent volume of distribution during the terminal phase (2,000–3,570 L) and a moderate to high apparent total body clearance (geometric mean [gMean] 758–1,430 mL/min) over the dose range 20–50 mg once daily [8]. The apparent gMean terminal elimination half-life is 37 h making it suitable for once-daily dosing [8]. After repeated dosing, there is at least twofold accumulation of afatinib based on area under the plasma concentration–time curve (AUC) or *C*_max_ estimates with no further accumulation in subsequent cycles [8]. Intake of high-fat/high-caloric food significantly decreased the bioavailability (AUC) of afatinib by ~39 % [9]; it is therefore recommended that patients take afatinib fasted [10]. Food intake should be avoided 3 h before and 1 h after afatinib administration. Afatinib undergoes minimal metabolism, which is governed by non-enzyme-catalysed adduct formation with proteins or nucleophilic small molecules, and is predominantly non-renally excreted via the entero-hepatic system [11]. Preclinical studies indicated that protein binding was likely to be moderate to high [12]. The in vitro metabolic profile and pharmacokinetic data suggest no interaction between afatinib and cytochrome P-450 substrates [11]. The highest dose intended for clinical use is 50 mg once daily.

In view of the biliary excretion and high plasma protein binding of afatinib, it is important to characterize the pharmacokinetics of afatinib in subjects with hepatic impairment. Here, we report data from a Phase I trial in healthy volunteers and hepatically impaired patient volunteers that was conducted to assess the impact of different degrees of hepatic impairment on the pharmacokinetics and safety of afatinib.

## Methods

This was a single-centre, open-label, single-dose, dose-escalation study (NCT01298063) using a matched-group design. The study protocol was approved by an independent ethics committee (Ethik-Kommission der Landesärztekammer Schleswig–Holstein, Bad Segeberg, Germany) and competent authority (BfArM, Bonn, Germany), and conducted in accordance with the Declaration of Helsinki. Written informed consent was obtained from all subjects before study entry.

### Subjects

Male or female non-smoker subjects aged 18–75 years and with a body mass index between 18.5 and 34.0 kg/m^2^ with chronic liver disease stable for at least 3 months were enrolled into two pre-specified groups: (a) mild hepatic impairment, Child-Pugh A (scores 5 or 6) [13], and (b) moderate hepatic impairment, Child-Pugh B (scores 7–9). Each individual was matched with a healthy control of the same gender, age (±10 years), weight (±10 %) and, if possible, creatinine clearance (±30 mL/min) according to the Cockcroft–Gault formula (Table 1). Healthy subjects were eligible based on the lack of clinically relevant history or physical examination and electrocardiography (ECG) findings, vital signs and clinical laboratory tests. Overall, creatinine clearance was >70 mL/min for healthy volunteers and >40 mL/min for subjects with hepatic impairment.

**Table 1:** Baseline characteristics of the study population included in pharmacokinetic analysis

	Mild hepatic impairment (*n* = 8)	Healthy matched controls (*n* = 8)^a^	Moderate hepatic impairment (*n* = 8)	Healthy matched controls (*n* = 8)^b^	Moderate hepatic impairment (*n* = 3)	All groups combined (*n* = 35)
Afatinib dose (mg)	50	50	50	50	30	30/50
Male [No. (%)]	6 (75.0)	6 (75.0)	5 (62.5)	5 (62.5)	3 (100)	25 (71.4)
Age (years)	53.9 ± 9.0	55.9 ± 12.6	54.8 ± 9.0	53.1 ± 7.9	64.3 ± 6.1	55.3 ± 9.5
White race [No. (%)]	8 (100)	8 (100)	8 (100)	8 (100)	3 (100)	35 (100)
Height (cm)	178.8 ± 8.4	175.3 ± 8.4	171.6 ± 9.7	173.0 ± 11.7	180.0 ± 1.7	175.1 ± 9.3
Body mass index (kg/m^2^)	27.1 ± 4.0	28.4 ± 3.9	26.8 ± 3.8	26.0 ± 2.0	25.1 ± 5.6	26.9 ± 3.6
Creatinine clearance (mL/min)	91.4 ± 20.3	101.0 ± 32.9	87.3 ± 22.6	90.8 ± 14.2	75.5 ± 8.8	91.2 ± 22.5

Results presented as mean ± SD unless otherwise stated

^a^Matched to subjects with mild hepatic impairment

^b^Matched to subjects with moderate hepatic impairment

Exclusion criteria in hepatic impaired subjects were severe cardiovascular disorders in the preceding 6 months; significant or recent acute gastrointestinal disorders; hepatic encephalopathy >grade 2 by number connection test; changes in chronic medication in the 4 weeks prior to trial drug administration (other than discontinuation of medications known to interact with P-glycoprotein); gastrointestinal bleeding in past 3 months; serum albumin <20 g/L; or haemoglobin <8 g/dL. For both patients and healthy controls, treatment with any medication with a half-life of >24 h in the month before or during the trial, potent P-glycoprotein inhibitors or inducers, or any medication known to interact with afatinib was prohibited. Women who were pregnant, breastfeeding or those of child-bearing potential not using adequate contraception for 3 months before and during the study were excluded.

### Study design and treatments

After an overnight fast (≥10 h), subjects received a single oral dose of afatinib (Boehringer Ingelheim Pharma GmbH & Co. KG, Germany) administered as a film-coated tablet with 240 mL of water in the sitting or standing position. Water was allowed ad libitum except 1 h before and 2 h after dosing, and standardized meals were served at least 2 h after dosing. Subjects were closely observed in the clinic for at least 24 h after dosing until discharge 48 h later and subsequently returned for follow-up blood sampling. Follow-up by telephone was arranged 28 days post-dosing.

### Pharmacokinetic evaluation

Blood samples were collected into potassium-EDTA-anticoagulant tubes before and 0.5, 1, 2, 3, 4, 5, 6, 7, 8, 9, 12, 24, 36, and 48 h after dosing, and then at 24-h intervals up to 10 days after dosing. All samples were centrifuged within 30 min of collection. A 15-mL sample for determination of plasma protein binding was also collected in 3 × 4.9 mL vials (monovettes coated with EDTA) on day 1 before dosing and centrifuged at 4,000 rpm for 10 min at 4 °C. All plasma samples were stored at −20 °C until analysis. Urine was collected in containers before and 0–4, 4–8, 8–12, 12–24, 24–48 and 48–72 h after dosing. To prevent adsorption losses of afatinib, a detergent (Tween 20) was added to the containers before urine collection. For each collection, the urine was weighed and homogenized and aliquots were stored at −20 °C until analysis.

Plasma and urine concentrations of the free base of afatinib (BIBW 2992 BS) were analysed using validated liquid chromatography-mass spectrometry (HPLC–MS/MS) methods (Nuvisan GmbH, Neu-Ulm, Germany) [11, 14]. Plasma concentrations within the validated concentration range were used to calculate the pharmacokinetic parameters. The calibration curves for afatinib covered the range 0.100–50.0 ng/mL for plasma and 5.00–1,000 ng/mL for urine. The lower limit of quantification of afatinib was 0.100 ng/mL in plasma and 5.00 ng/mL in urine. Assay performance was assessed by back calculation of calibration standards, tabulation of the standard curve fit function parameters and measurement of quality control samples. For HPLC–MS/MS bioanalysis of afatinib plasma and urine concentrations, assay accuracy [deviation from nominal value (%)] and precision [coefficient of variation (%)] of quality control samples spiked at three concentrations were between 3.9 and 9.6 % for plasma, and between 4.9 and 8.0 % for urine, respectively.

In vitro plasma protein binding of afatinib was determined in pre-dose plasma samples after spiking of 150 nmol/L (=72.9 ng/mL) [^14^C]-radiolabeled afatinib using equilibrium dialysis (Covance Laboratories, Harrogate, UK). Previous in-house studies demonstrated the suitability of this methodology (unpublished data on file, Boehringer Ingelheim, Biberach, Germany).

### Safety evaluation

The safety of afatinib was assessed by 12-lead ECG, vital signs (pulse, blood pressure), routine laboratory assessments, adverse event reporting and assessment of global tolerability by the investigator. Adverse events were graded according to the National Cancer Institute Common Terminology Criteria for Adverse Events (CTCAE) version 3.0.

**Fig. 1:**
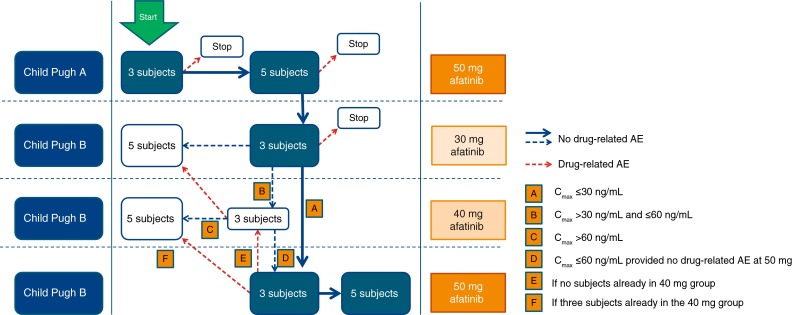
Planned inclusion and dosing progression for hepatic impaired subjects according to assessment of adverse events (AEs) and exposure (*C*_max_) for afatinib measured by interim pharmacokinetics. AE adverse event. The *bold lines* indicate the trial pathway if there was no drug-related toxicity and plasma exposure (*C*_max_) did not exceed defined thresholds. *Dotted lines* indicate stopping points if drug-related toxicity was observed. *Thin lines* indicate alternative trial pathways. The flow chart does not display the inclusion of matched healthy subjects

For safety reasons, individuals with hepatic impairment were to be dosed in a 3 + 5 design, with three patients being treated at the initial dose (Fig. 1). The decision to proceed with further afatinib administrations at higher doses was based on a combined assessment of drug-related adverse events and *C*_max_ (gMean) of afatinib by interim pharmacokinetic measurements after dosing of three subjects in each hepatic impairment group and their respective controls. Subjects with mild hepatic impairment were initially dosed with afatinib 50 mg; if no subject had a drug-related adverse event of CTCAE grade ≥3, or <2 subjects had a drug-related adverse event of CTCAE grade ≥2 and *C*_max_ was ≤30 ng/mL, three patients with moderate hepatic impairment were then dosed with 30 mg afatinib. If no drug-related toxicity was observed, the remaining five subjects with moderate hepatic impairment received 50 mg afatinib or an interim dose of 40 mg if *C*_max_ was >30 ng/mL and ≤60 ng/mL. These pharmacokinetic thresholds took into consideration that in a trial of healthy subjects receiving single doses of afatinib (20, 30, 40 or 50 mg), individual *C*_max_ values exceeding 60 ng/mL were well tolerated [15]. Further, in a trial of patients with solid tumours who received afatinib 30 and 45 mg once daily for 14 days, no drug-related adverse events of CTCAE grade >2 were reported and individual *C*_max_ values after the first dose (day 1) did not exceed 100 ng/mL [14]. To allow for additional safety monitoring, staggered dosing was preferred in each of the subgroups of five subjects (three subjects on 1 day, followed by two subjects on the following day). Afatinib 50 mg was used in this study because it is the maximum tolerated dose and is the highest dose for therapeutic use.

### Statistical analyses

The study planned to recruit up to 38 subjects (see Fig. 1) with the aim of entering eight subjects with mild liver impairment (at 50 mg afatinib), eight subjects with moderate liver impairment (at either 30, 40 or 50 mg afatinib) and eight healthy matched controls to each of this two groups (in total 16 healthy subjects). A total of 32 subjects (eight per group) receiving 50 mg afatinib for the primary analysis were judged an adequate sample size, in agreement with regulatory guidance of pharmacokinetic studies in patients with impaired hepatic function [16].

The primary pharmacokinetic endpoints were AUC from time zero extrapolated to infinity (AUC_0**–**∞_) and *C*_max_. AUC from 0 to the time of the last quantifiable data point (AUC_0–tz_) was a secondary endpoint. The log-transformed AUC and *C*_max_ values for afatinib were compared between groups using an analysis of variance (ANOVA) model with ‘hepatic status’ as fixed effect and ‘matched pair’ as random effect. The least square means and 90 % confidence intervals (CI) based on the t-distribution were calculated and then back-transformed to the original scale to provide gMean and interval estimates for the ratio between response under test (liver impaired subjects) and response under reference (healthy subjects). For all other parameters, descriptive statistics were presented. Non-compartmental analysis of plasma concentration–time data was performed using WinNonlin^®^ Professional Network version 5.2 software (Pharsight Corporation, Cary, NC, USA). Statistical analyses were performed using SAS^®^, version 9.2 (SAS Institute Inc., Cary, NC, USA).

## Results

Thirty-five subjects were enrolled and completed the study (16 healthy subjects, 8 subjects with mild hepatic impairment and 11 with moderate hepatic impairment). Thirty-two subjects received a single dose of 50 mg afatinib, and three received a single 30 mg dose. Primary analysis was based on 32 subjects who received the 50 mg dose (Table 1). All 35 patients were included in the assessment of safety.

### Pharmacokinetics of afatinib

**Fig. 2:**
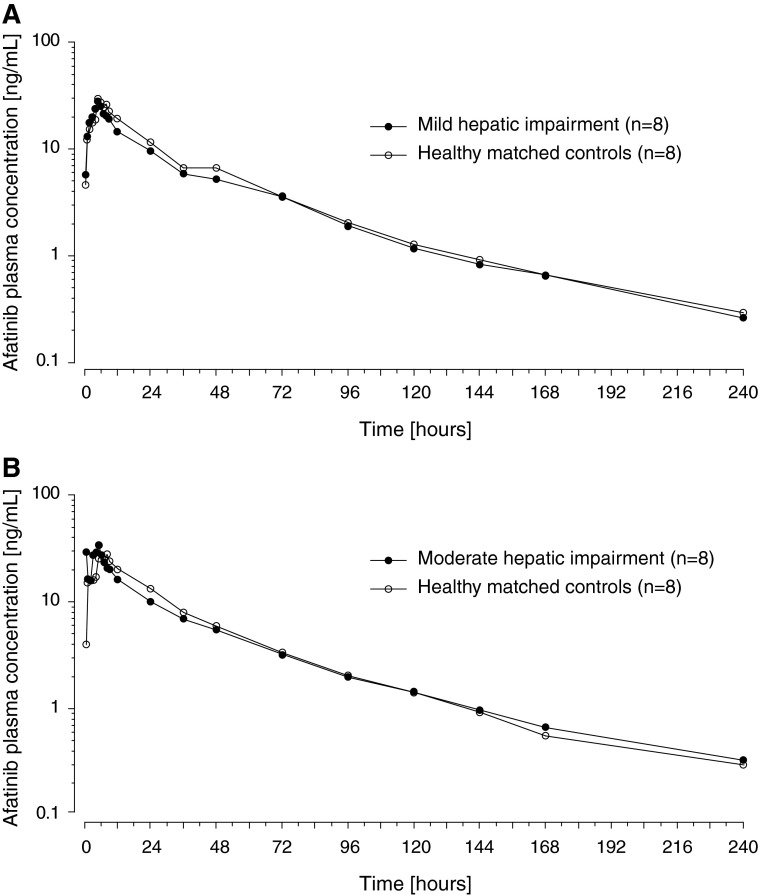
Geometric mean plasma concentration–time profiles of single-dose afatinib 50 mg in subjects with **a** mild and **b** moderate hepatic impairment compared with matched healthy controls (semi-log scale)

There were no notable differences in the plasma concentration–time profiles between subjects with mild or moderate hepatic impairment and their matched healthy controls (Fig. 2). Afatinib showed a biphasic disposition profile, and a long terminal phase was detected extending out to the last sampling point of 240 h. The extent of exposure as indicated by AUC_0–∞_ and *C*_max_ was generally comparable between the matched treatment groups (Table 2). For subjects with mild hepatic impairment, the adjusted gMean ratio for AUC_0**-**∞_ was 92.6 % (90 % CI 68.0–126.3 %) and for *C*_max_ was 109.5 % (90 % CI 82.7–144.9 %), compared with healthy control subjects. For subjects with moderate hepatic impairment, the corresponding adjusted gMean ratios were 94.9 % (90 % CI 72.3–124.5 %) and 126.9 % (90 % CI 86.0–187.2 %), respectively (Table 3).

**Table 2:** Geometric mean pharmacokinetic parameters after a single dose of 50 mg afatinib for subjects with mild or moderate hepatic impairment and matched healthy controls

Parameter and unit	Mild hepatic impairment (*n* = 8)	Matched controls to mild hepatic impairment (*n* = 8)	Moderate hepatic impairment (*n* = 8)	Matched controls to moderate hepatic impairment (*n* = 8)
Primary endpoints				
AUC_0–∞_ (ng h/mL)	886 (53.7)	956 (22.7)	934 (31.0)	985 (32.3)
*C*_max_ (ng/mL)	33.7 (51.7)	30.7 (33.7)	39.5 (40.1)	31.1 (46.0)
Secondary endpoint				
AUC_0–tz_ (ng h/mL)	842 (50.8)	930 (22.5)	904 (31.4)	956 (33.3)
Other endpoints				
*t*_max_^a^ (h)	5.0 (0.5–8.0)	5.0 (3.0–7.0)	4.0 (0.5–5.0)	7.5 (5.0–9.0)
* t*_1/2_ (h)	74.9 (47.6)	60.3 (14.9)	64.3 (13.1)	59.9 (28.5)
Ae_0–72_ (mg)	1.29 (40.5)	1.21 (14.3)	1.04 (47.7)	0.998 (26.6)
fe_0–72_ (%)	2.58 (40.5)	2.43 (14.3)	2.07 (47.7)	2.00 (26.6)
CL_R,0–72_ (mL/min)	32.7 (37.6)	27.2 (26.4)	24.1 (71.0)	21.5 (32. 8)

*Ae*_*0*–*72*_ amount of unchanged drug excreted into the urine over 72 h, *AUC*_*0*–*tz*_ area under the drug plasma concentration–time curve from time 0 to the time of the last quantifiable data point, *AUC*_*0*–*∞*_ area under the drug plasma concentration–time curve from time 0 to infinity, *C*_*max*_ maximum drug concentration in plasma, *CL*_*R,0*–*72*_ renal clearance over 72 h, *CV* *%* coefficient of variation (%), *fe*_*0*–*72*_ fraction of oral dose observed in urine over 72 h, *t*_*1/2*_ terminal elimination half-life, *t*_*max*_ time to reach *C*_max_

Results are presented as geometric mean (geometric CV %) unless stated otherwise

^a^Median and range

**Table 3:** Adjusted gMean ratios for AUC and *C*_max_ of afatinib 50 mg (subjects with hepatic impairment versus controls), % with 90 % CI

Parameter and unit	Hepatic impairment group	gMean ratio (%) (90 % CI)^a^	Intraindividual gCV (%)^b^
AUC_0–∞_ (ng h/mL)	Mild	92.6 (68.0–126.3)	33.6
	Moderate	94.9 (72.3–124.5)	31.6
*C*_max_ (ng/mL)	Mild	109.5 (82.7–144.9)	30.3
	Moderate	126.9 (86.0–187.2)	42.8
AUC_0–tz_ (ng h/mL)	Mild	90.6 (66.9–122.7)	32.8
	Moderate	94.5 (71.6–124.8)	32.4

*AUC*_*0*–*tz*_ area under the drug plasma concentration–time curve from time 0 to the time of the last quantifiable data point, *AUC*_*0*–*∞*_ area under the drug plasma concentration–time curve from time 0 to infinity, *C*_*max*_ maximum drug concentration in plasma, *gCV* geometric coefficient of variation (%), *gMean* geometric mean

^a^Ratio of gMeans (hepatic impairment subjects to healthy subjects). Statistical assessment of differences in pharmacokinetic parameters between patients with mild and moderate hepatic impairment and healthy subjects was performed using separate ANOVA models

^b^See Table 2 for the individual group means for each treatment group

For subjects with mild hepatic impairment, median time to peak plasma concentration (*t*_max_) was the same as matched healthy controls (5 h). For subjects with moderate hepatic impairment, *t*_max_ occurred earlier than for matched healthy controls (4.0 h for moderate impairment vs. 7.5 h for healthy controls). The range of *t*_max_ values was also larger in subjects with hepatic impairment compared with matched healthy controls; for mild impairment, values were between 0.5 to 8 h versus 3 to 7 h for matched controls, and for moderate impairment, values were between 0.5 to 5 h, versus 5 to 9 h for matched controls. The gMean terminal half-life ranged from 60 to 75 h and was comparable for subjects with hepatic impairment and normal hepatic function (Table 2).

**Fig. 3:**
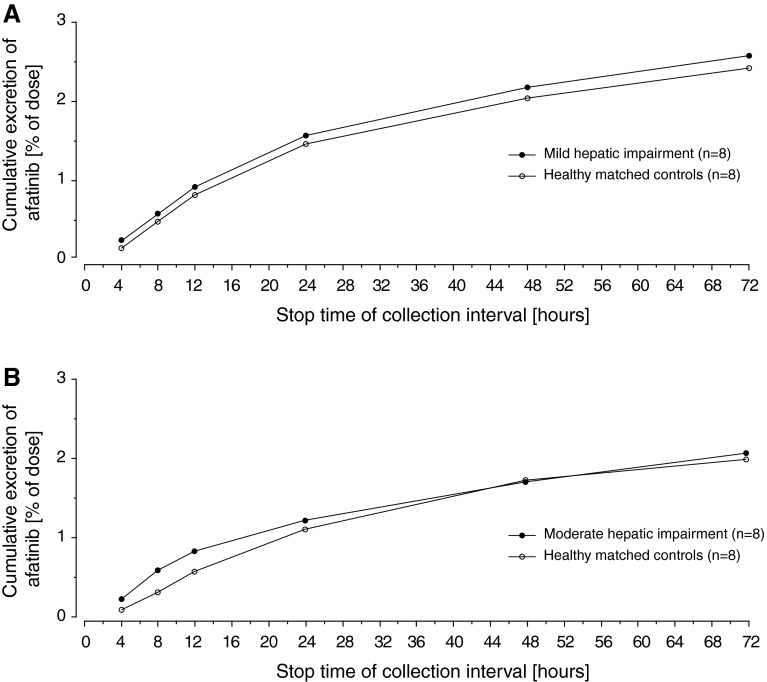
Geometric mean cumulative urinary excretion of afatinib (%) after single-dose afatinib 50 mg in subjects with **a** mild and **b** moderate hepatic impairment compared with matched healthy controls

There were quantifiable urinary concentrations of afatinib over the entire sampling interval (up to 72 h post-dose) in all subjects. The total cumulative fraction of afatinib excreted in the urine (fe_0–72_) in subjects with hepatic impairment was generally low and comparable with matched controls (gMean values between 2.0 and 2.58 %; Table 2). The gMean excretion profiles showed no noteworthy differences between the treatment groups (Fig. 3).

The arithmetic mean ± SD fraction of [^14^C] afatinib (target concentration 72.9 ng/mL) bound to plasma proteins in pre-dose plasma samples was 94.6 ± 0.7 % in healthy controls (*n* = 16), 94.1 ± 1.1 % in subjects with mild hepatic impairment (*n* = 8) and 93.7 ± 0.7 % in subjects with moderate hepatic impairment (*n* = 11, three subjects that received afatinib 30 mg and eight subjects that received afatinib 50 mg). The overall mean percentage protein binding was 94.2 ± 0.9 %.

### Safety and tolerability

Single-dose afatinib 50 mg was well tolerated with few adverse events. None of the subjects experienced serious adverse events or discontinued the study due to an adverse event. Adverse events were reported in five (26 %) subjects with hepatic impairment (three mild, two moderate) and one (6 %) healthy control subject. Three patients with mild hepatic impairment (50 mg afatinib) had adverse events that were considered treatment-related. One of these subjects had a grade 3 lipase elevation; however, cholecystolithiasis with sludge phenomenon was observed on abdominal ultrasound of this subject, suggesting that this was the most likely cause of the increase. This subject had a similar episode of asymptomatic lipase increase prior to enrolment in the clinical trial. The other two treatment-related events were grade 2 headache and nausea in one subject and grade 1 diarrhoea in one subject. All adverse events had resolved by the end of the trial. There were no other clinically relevant changes in laboratory parameters, vital signs or ECG.

## Discussion

Following administration of a single dose of 50 mg afatinib, exposure levels were comparable for subjects with mild or moderate hepatic impairment and healthy controls with normal liver function. The pharmacokinetic characteristics of afatinib either in healthy subjects or in subjects with mild/moderate hepatic impairment within this trial were found to be consistent with the pharmacokinetic characteristics of afatinib in previous trials of cancer patients with various advanced solid tumours [8]. Thus, mild or moderate hepatic impairment has no relevant influence on the pharmacokinetics of afatinib. This result is important since afatinib is preferentially eliminated by the entero-hepatic system.

The most likely reason why afatinib exposure is not increased in subjects with hepatic impairment may relate to the pharmacokinetic properties of the drug. Afatinib exhibits moderate to high plasma protein binding (mean fraction bound 94.6 ± 0.7 % at a concentration of 72.9 ng/mL in healthy volunteers). Thus, only a small fraction of the total plasma concentration is directly exposed to hepatic metabolism and excretion. Metabolism of afatinib is negligible [11], resulting in biliary excretion of predominantly unchanged afatinib. Afatinib is a P-gp substrate, and unchanged afatinib may be excreted in the liver via P-gp in the bile. However, hepatic impairment is not known to influence P-gp expression in the liver meaning that mild to moderate hepatic impairment is not likely to substantially affect the biliary elimination process of afatinib.

Hepatic impairment had no clinically relevant effect on the absorption, distribution or elimination of afatinib. The terminal half-life of afatinib in all treatment groups was similar although the gMean value was about twice that previously reported [8] (60–75 h in the current study versus ~37 h). This was most likely due to the longer sampling period (240 h) in this trial compared with that in other trials (24 or 72 h) [8]. Additionally, the lower limit of quantification for afatinib in this study (0.1 ng/mL) was lower than that in previous studies (0.5 ng/mL). It is possible that the observed prolonged elimination may be attributable to circulating covalent adducts, or non-covalent binding in deep compartments, given that afatinib is a basic compound with high lipophilicity and a large volume of distribution. However, it is notable that covalent binding accounted only for 0.02 % of the dose 48 h after dosing in a metabolism study in healthy volunteers [11]. Thus, it is considered unlikely that covalent binding may act as a relevant storage compartment of afatinib. Furthermore, gMean half-life after multiple dosing was around 37 h (plasma concentration measured until 72 h) [8], which is consistent with 2.8-fold accumulation based on AUC [17]. Taken together, these observations suggest that the extended terminal elimination phase when sampling up to 240 h after dosing was not relevant for the accumulation observed after multiple dosing and can therefore be considered negligible.

Renal excretion was similar in healthy and hepatic impaired subjects and was generally low in all treatment groups. Although this was a single-dose study, the conclusions based on exposure (*C*_max_ and AUC_0**–**∞_) data are likely to be valid under steady-state conditions, in that single-dose data predict multiple-dose exposure [8].

The current analysis showing that mild and moderate hepatic impairment had no obvious effect on the pharmacokinetics of a single afatinib dose is consistent with data from a population pharmacokinetic analysis of patients with advanced solid tumours receiving continuous doses of afatinib [18]. In this analysis, hepatic impairment, either based on individual clinical laboratory tests (alanine transaminase, aspartate transaminase and bilirubin) or a composite liver dysfunction measure, had no significant influence on afatinib exposure. Limited data in the dataset did not allow robust assessment of moderate hepatic impairment, and there were no data for patients with severe hepatic impairment.

A single dose of afatinib 50 mg was well tolerated by most subjects. All adverse events were of mild to moderate intensity. Asymptomatic lipase increases have been previously reported following one or two doses of afatinib 40 mg co-administered with multiple doses of ritonavir [19]. The overall frequency of adverse events was higher in subjects with hepatic impairment compared with healthy subjects, which may be due to the poorer health status of these subjects. However, the proportion of subjects reporting adverse events was in keeping with previous healthy volunteer trials [11, 19].

In conclusion, there were no clinically relevant differences in exposure (*C*_max_ and AUC_0**–**∞_) for subjects with mild or moderate hepatic impairment compared with matched healthy controls after a single dose of afatinib 50 mg. Based on these data, adjustments to the starting dose of afatinib are not considered necessary for patients with mild or moderate hepatic impairment. As systemic exposure to afatinib was not assessed in patients with severe (Child-Pugh C) hepatic dysfunction, no recommendations can be made for this group of patients.
